# Mechanisms influencing network topology in plant–hummingbird pollination networks

**DOI:** 10.1098/rspb.2025.2249

**Published:** 2025-11-26

**Authors:** Ricardo Sánchez-Martín, Elisa Barreto, Melina F. Maxwell, Francois Duchenne, Holger Beck, Rafaela Bobato, Emanuel Brenes, Daniela Bôlla, Nicole Büttner, Ana Paula Caron, Alejandro Castro Jiménez, Nelson Chaves-Elizondo, María José Gavilanes, Anna Sofia Görlich, Esteban A. Guevara, Miriam Kaehler, Tiago Machado de Souza, Miguel Machnicki-Reis, Andrés Sebastián Marcayata-Fajardo, Cauã Galeazzi de Menezes, Andrea Nieto, Rafael Oliveira, Ricardo Augusto Camargo de Oliveira, Alejandro Restrepo-González, Friederike Richter, Bryan Gastón Rojas, Luciele Leonhardt Romanowski, Romulo Silva Cícero Silva, Wellinton Luiz de Souza, Francisco Tobar, Danila Syriani Veluza, Rafael O. Wüest, Thais Bastos Zanata, Krystal Zuniga, Tatiana Santander, María A. Maglianesi, Isabela G. Varassin, Catherine H. Graham

**Affiliations:** ^1^ Department of Biodiversity and conservation biology, Swiss Federal Institute for Forest Snow and Landscape Research WSL, Birmensdorf 8903, Switzerland; ^2^ Laboratório de Macroecologia e Conservação Marinha, Universidade Federal de Santa Maria, Santa Maria, Rio Grande do Sul 97105-340, Brazil; ^3^ Estacion Biologica de Donaña (CSIC), Seville 41013, Spain; ^4^ Santa Lucia Cloud Forest Reserve, Nanegal, Quito 079414, Ecuador; ^5^ Laboratório de Interações e Biologia Reprodutiva, Universidade Federal do Paraná, Curitiba, Paraná 80020-300, Brazil; ^6^ Royal Botanical Gardens, Kew, London, TW9, UK; ^7^ Escuela de Ciencias Exactas y Naturales, Universidad Estatal a Distancia, San Jose, San José 474-2050, Costa Rica; ^8^ National Institute for Amazonian Research (INPA), Manaus, Amazonas 69067-375, Brazil; ^9^ Un poco del Chocó - Reserve and Biological Station, Gualea, Quito 170184, Ecuador; ^10^ Museu de História Natural Capão da Imbuia – MHNCI, Curitiba, Paraná 82810-00, Brazil; ^11^ Universidad de Costa Rica, San Jose, San José 11501-2060, Costa Rica; ^12^ Universidad San Francisco de Quito, Quito, Pichincha 170901, Ecuador; ^13^ Department of Biology, Conservation Ecology, Philipps-Universität Marburg, Marburg, Hessen 35043, Germany; ^14^ Mater Natura - Instituto de Estudos Ambientais, Curitiba, Paraná 80010-050, Brazil; ^15^ Instituto Nacional de Biodiversidad, Quito, Pichincha 170506, Ecuador; ^16^ Senckenberg Biodiversity and Climate Research Centre, Frankfurt, Hessen 60325, Germany; ^17^ Botanik, Staatliches Museum für Naturkunde Stuttgart (SMNS), Stuttgart, 70191, Germany; ^18^ Stiftung Naturschutz Berlin, Berlin, Berlin 10785, Germany; ^19^ Universidad del Azuay, Cuenca, Azuay 010204, Ecuador; ^20^ Aves y Conservación/BirdLife in Ecuador, Quito 170509, Ecuador; ^21^ Laboratório de ecologia humana e etnobotânica, Universidade Federal de Santa Catarina, Florianópolis, Santa Catarina 88010-970, Brazil; ^22^ Laboratório de Interações e Síntese em Biodiversidade, Departamento de Botânica e Ecologia, Instituto de Biociências, Universidade Federal de Mato Grosso, Cuiabá, Mato Grosso 78060-900, Brazil

**Keywords:** connectance, elevation gradients, functional diversity, modularity, nestedness, network structure, plant–hummingbird interactions, pollination networks, specialization, trait matching

## Abstract

Ecological communities result from complex species interactions, often summarized in interaction networks. The structure of these networks is described by metrics that provide insight into community assembly, ecosystem functioning and coevolutionary processes. Despite advances in measuring and mapping network structure, the mechanisms underlying its formation remain less explored. Network metrics may vary across communities owing to changes in species diversity and environmental conditions. However, network metrics may remain invariant if mechanisms influencing interactions (linkage rules) are independent of species composition and environmental conditions and instead influenced by traits. We investigated whether changes in taxonomic, phylogenetic and functional diversity along elevation gradients influence network modularity, nestedness, connectance and specialization across 32 sites in Brazil, Costa Rica and Ecuador. Despite elevation’s impact on diversity, we found that it had no effect on network structure, which remained consistent across elevations. Instead, trait-based mechanisms, specifically the matching between hummingbird bills and flower corollas, emerged as a consistent driver of network structure. Species showing strong trait matching contributed more to modularity and specialization, but less to nestedness and connectance than expected by chance. These results suggest that trait matching influences the invariant structure of plant–hummingbird networks, persisting despite shifts in diversity across biogeographical regions and elevational gradients.

## 1. Introduction

Mutualistic networks, which describe mutually beneficial interactions among species [1], provide insights into community assembly, ecosystem functioning, coevolutionary processes and the maintenance of biodiversity [2–6]. While many studies explore how community network structure varies across environmental gradients [7–9], others focus on inferring the mechanisms underlying species’ interactions (often referred to as linkage rules [10]), such as trait matching [11–13]. However, a key gap remains: the extent to which linkage rules influence emergent network structure. If species-level linkage rules consistently shape network structure, then network properties should remain stable despite changes in diversity across gradients. Establishing this connection would yield insight into the mechanisms influencing network structure across diversity gradients.

The network structure of communities may vary as a result of changes in diversity across environmental gradients. Higher taxonomic diversity is often associated with reduced connectance [14,15] and increased specialization [16]. Phylogenetic diversity, which captures variation in evolutionary history, has been associated with highly nested networks [17]. Functional diversity, reflecting variation in traits within a community, has been positively associated with both modularity and specialization [18,19]. In contrast, if trait-based linkage rules dictate interactions among species, network structure could remain consistent across diversity gradients. For example, the structure of ant–plant mutualistic networks [20] and Antarctic lichen mycobiont–photobiont networks [21] remained similar despite changes in the composition and richness of interacting species along spatial gradients. Similarly, modularity in plant–insect pollinator networks did not vary across space [22] or time [23].

It is widely recognized that plant–hummingbird interactions are often structured based on the similarity between hummingbird bill lengths and plant corolla lengths (i.e. trait matching; [11–13,24]). Trait matching improves hummingbird handling efficiency and enhances plant fitness [25–27]. However, hummingbirds can also feed on flowers with tube lengths that differ significantly from their bill morphology [28,29]. They change their feeding preferences depending on resource availability and competition with other hummingbirds [26,30,31], suggesting that local context may also play a role in determining species' roles within the network. By examining how much each species depends on trait matching and how this dependence shapes species-level contributions to network structure, we move beyond pattern description to uncover the mechanisms underlying network structure.

Understanding whether trait matching consistently contributes to network structure across different contexts requires datasets that capture variation along spatial gradients. Elevational gradients provide the opportunity to study network shifts in response to environmental changes within a region [8]. Studying elevational gradients across different regions, each with distinct species pools and evolutionary histories, enables us to identify general patterns in network structure. However, such comparisons are often hindered by a lack of large-scale, systematically sampled datasets that incorporate both local and regional variation. Macroecological studies address this gap by combining local network data or using metaweb approaches. While these approaches are valuable, each has limits: local network compilations often vary in sampling effort and procedures [32,33], and metawebs assume that co-occurring species interact [34], simplifying complex interactions into binary relationships that may overlook factors like species abundances, temporal overlap and spatial context [35]. We overcome these limitations by using a systematically sampled dataset of 32 plant–hummingbird networks collected with a consistent method and an unprecedented sampling effort of two years per network [36], providing a robust database for testing hypotheses about plant-pollinator network structure across elevational gradients and among regions.

Here, we explore the consistency of network metrics in 32 plant–hummingbird pollination networks along elevational gradients in Brazil, Costa Rica and Ecuador. We summarize network structure by calculating modularity, nestedness, connectance and specialization. We test whether these metrics change across the elevation gradient and among regions. In parallel, we assess changes in taxonomic, phylogenetic and functional diversity along the same gradients. We hypothesize that if trait matching plays a key role in structuring networks, then sites should exhibit low variation in network structure metrics across elevational gradients in the different regions, despite diversity shifts occurring along these gradients. To test the relationship between network structure and trait matching, we assess whether species-level contributions to network metrics vary as a function of their contribution to trait matching. We expect that species relying on trait matching would contribute positively to modularity and specialization, as well-matched species tend to form clusters. This compartmentalization, in turn, reduces connectance by limiting interactions among modules. Nestedness may also be low since specialist hummingbirds rarely interact with the plants visited by generalists [3]. Uncovering the generality of trait-based mechanisms in determining network structure across diverse contexts provides deeper insight into network topology and thus could support trait-informed monitoring and conservation.

## 2. Methods

### (a) Network data

**Figure 1.**
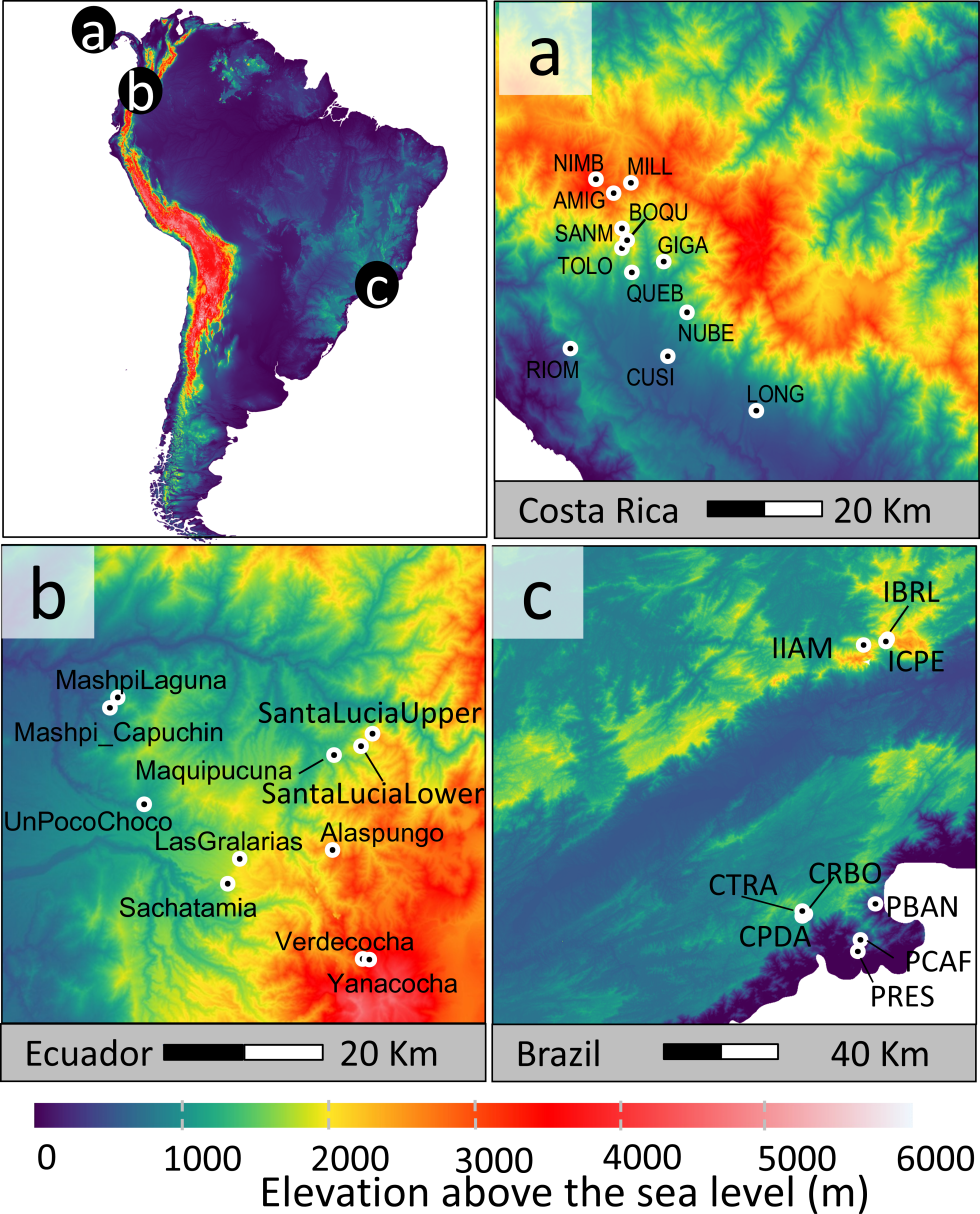
Map displaying the location of the 32 sites where interaction data were collected.

We studied 32 plant–hummingbird networks across Latin America (figure 1). In Costa Rica, we sampled 12 networks along the Pacific slope of the Talamanca Mountain Range (636–3224 m a.s.l.). In Ecuador, we sampled 11 networks across the Chocó region in Pichincha Province, on the western slopes of the Andes (776–3522 m a.s.l.). In Brazil, we sampled 9 networks in the southeastern Atlantic Forest (10–2271 m a.s.l.) in Serra da Bocaina and Serra da Mantiqueira. Each location featured a 1.5 km × 10 m transect. Transects were sampled once a month for approximately 2 years between February 2017 and December 2022 (see electronic supplementary material Annex I, table S1 for country-specific sampling periods and estimates of sampling completeness). Each month, we identified all flowering plant species within 5 m on each side of the transect. To capture the broad feeding niche of hummingbirds [31,37], we not only sampled plants with a hummingbird pollination syndrome [38] but also those that lacked the syndrome and were observed being visited by hummingbirds during our extensive fieldwork. To conduct the extensive sampling of interactions in a replicable way across time and space, we documented plant–hummingbird interactions using time-lapse cameras. Each month, we selected 12 focal plant individuals to monitor, prioritizing species richness and representing the relative availability of floral resources at that time. Candidate plants were monitored using six ‘Plotwatcher Pro’ time-lapse cameras, positioned at 80–100 cm from open flowers, capturing one picture per second for 3 days. After the first three days, the cameras were moved to the remaining six plants. We avoided deploying the cameras too close together by distributing them along the transect wherever possible, ensuring a minimum spacing of 20 m between cameras. We used DeepMeerkat software to identify potential frames with hummingbirds [39]. These frames were manually reviewed to identify the species. We excluded records where hummingbirds were robbing the nectar through holes in the floral structures. Moreover, if a plant and hummingbird species co-occurred at the same site and time but were not recorded interacting legitimately, we assumed they did not interact. Finally, we aggregated interactions by site and month. The interaction dataset included 54 662 interactions detected between 70 hummingbird species and 424 plant species. Summary statistics for each country are provided in electronic supplementary material Annex I, table S1.

### (b) Trait data

We collected flowers from each plant species (mean: 9; range: 1–49, s.d. = 7). We measured the corolla length (the straight-line distance between the base of the corolla and the separation of petals), corolla opening (the narrowest distance between the extremes of the corolla opening) and corolla curvature (the inverse radius of the circle fitted to the midpoint of the flower, following [27]). Plant traits were measured on standardized pictures using the software ImageJ v. 1.53 [40]. We used the mean trait values for our analyses because traits were not collected at specific sites or times, and thus we could not estimate spatial or temporal intraspecific variation. For hummingbirds, we gathered data on bill length (the primary trait related to trait matching), as well as body mass, tail length, wing chord and tarsus length—traits associated with manoeuvrability and hovering capacity, which may influence visitation patterns [41,42]. Hummingbird trait data were taken from [43–45].

### (c) Diversity metrics

We calculated taxonomic, phylogenetic and functional diversity for each site and trophic level (plants and hummingbirds). Taxonomic diversity was calculated as the number of unique species present in each community. Phylogenetic diversity was computed as the standardized effect size of phylogenetic diversity (Faith’s PD) using the function ‘ses.pd’ from the R package 'picante' [46]. See electronic supplementary material Annex I, Supporting Methods, for details on phylogenetic tree construction. We used functional divergence as a measure of functional diversity, which quantifies the degree to which the abundance of a community is distributed towards the extremities of occupied functional trait space [47,48]. We used corolla length, opening and curvature for the functional divergence of plants. Hummingbirds’ functional divergence was calculated based on bill length, body mass, tail length, wing chord and tarsus length. Functional divergence was calculated using the ‘dbFD’ function in the R package FD [47].

### (d) Community-level network metrics

We built a quantitative interaction matrix for each community, with link weights representing visitation rates (number of visits per hour) for each hummingbird–plant species combination over the total sampling time. To define the structure of the networks, we calculated four community-level descriptors. (i) Modularity (Q) quantifies how well a network is divided into modules, with higher values indicating stronger compartmentalization. (ii) Connectance (C) represents the proportion of realized links relative to possible links. (iii) Community specialization (H2’) quantifies how much observed interactions deviate from expectations, with higher values indicating greater selectiveness. (iv) Nestedness (WNODF) describes the tendency of specialists to interact with generalists. We quantified modularity (Q) using the Beckett algorithm [49] with the ‘computeModules’ R function. We calculated connectance (C; [50]), specialization (H2’; [51]) and nestedness (WNODF; [52]) using the ‘networklevel’ function. All functions for network descriptors are available in the ‘bipartite’ R package [53].

We standardized network metrics using *z*-scores, comparing observed values with the mean and standard deviation of a null model that accounted for flower phenology, hummingbird abundance and plant sampling effort. Specifically, (i) we reshuffled hummingbird–plant interactions monthly to account for phenological overlap—that is, interactions were only possible when hummingbirds and plants co-occurred during flowering. (ii) The reshuffling also preserved each hummingbird species' total number of visits, maintaining their relative abundance. Since hummingbird abundance data came from camera recordings, they may not fully reflect actual community abundance. (iii) Finally, visits were allocated proportionally to each plant’s recording time, ensuring that plants that were filmed for longer received more visits. *Z*-scores were computed from 1000 null simulations, sufficient to ensure stable estimates across sites and metrics (electronic supplementary material Annex I, figure S1). A more liberal null model, lacking temporal constraints, produced similar results. However, we retained the temporally structured approach to avoid generating interactions between species that do not co-occur in time.

### (e) Species-level contribution to network metrics

Within networks, species contribute differently to network structure [54,55]. To determine the contribution of each species (i.e. node) to community network structure, we followed Saavedra *et al.* [56]. We recalculated each community network metric 1000 times after randomizing only the visits in which the focal species was involved. We then computed a *z*-score by comparing the observed community-level metric (with the focal species’ real interactions) with the distribution of metrics from the randomized networks. This *z*-score quantifies the extent to which the focal species contributes to a given network property compared with a null expectation. We chose this approach instead of calculating species-level metrics (i.e. d’, degree, species’ role in the network, among many others) because we were specifically interested in evaluating the individual species’ contributions to modularity, nestedness, connectance and specialization using a common approach.

The baseline reshuffling for *z*-score calculation was performed to control for phenological overlap, preserve hummingbird relative abundances and account for differences in plant sampling efforts, consistent with the approach used for community-level reshuffling, but for each node (i.e. species). For each focal hummingbird species, visits were allocated proportionally to the recording time of each plant (row-wise reshuffling). For each focal plant species, we reassigned the same number of visits based on the relative abundance of each hummingbird species during each month (column-wise reshuffling). Then, visitation rates were recalculated for the whole community. The new interaction matrix only altered rates for the row or column corresponding to the focal species, while all other rates remained unchanged from the original matrix. The species-level contributions to each metric are the *z*-scores of these metrics. Therefore, a positive contributor to a given community metric will have *z*-scores greater than 0, and a negative contributor will have *z*-scores smaller than 0.

### (f) Standardized effect of trait mismatch

To evaluate whether species-level contributions to network structure were influenced by trait matching, we measured how much the interactions of each focal species (both plants and hummingbirds) relied on trait matching. We calculated the observed trait mismatch for each focal species in each site. Trait mismatch is the average of the absolute differences between the pair-wise bill lengths of hummingbirds and corolla lengths of plants weighted by their visitation rate in each site. A trait-mismatch value of 0 indicates perfect matching between the traits of the focal species and its interaction partners. Higher values indicate increasing mismatches between them. The trait mismatch, TM, can be calculated for each hummingbird species (equation 2.1) or plant species (equation 2.2), using the following formulae:


(2.1) $$TM_{ik}=\frac{\sum_{jk}|bill\_length_{i}-Corolla\_length_{j}|*rate_{ijk}}{\sum_{jk}rate_{ijk}},$$

(2.2) $$TM_{jk}=\frac{\sum_{ik}|bill\_length_{i}-Corolla\_length_{j}|*rate_{ijk}}{\sum_{ik}rate_{ijk}},$$

where *i* refers to a given hummingbird species, *j* to a given plant species, *k* represents the site (or local network), and *rate_ijk_* is the visitation rate between hummingbird species *i* and plant species *j* within the local network *k*. We evaluated the deviation of the observed values of trait mismatch from random expectations based on 1000 simulations, using the same reshuffling procedure described for species-level contribution to network metrics, but applied to trait mismatch. Negative *z*-scores for trait mismatching indicate a stronger reliance on trait matching. In contrast, positive *z*-scores for trait mismatching indicate that species interact with highly mismatched partners.

### (g) Statistical analyses

#### (i) Effect of elevation on diversity

We used generalized linear models (GLMs) to test whether hummingbird and plant diversity varied with elevation and whether these patterns were consistent across countries. We used diversity metrics (taxonomic, phylogenetic or functional) as response variables and elevation as a predictor. Elevation was included as a linear and quadratic predictor to capture potential nonlinear effects. We included elevation in interaction with country (elevation × country) to assess whether elevation-related changes in network structure varied by country. If the interaction was not significant, we simplified the model to include only additive terms (elevation + country).

#### (ii) General patterns in community-level network structure

To evaluate general patterns in the structure of the networks, we conducted a *t*‐test for each *z*-score of the network metrics (modularity, nestedness, connectance and specialization). Additionally, to assess differences among countries for each network metric, we conducted Kruskal–Wallis tests separately for the network *z*-score metrics. If Kruskal–Wallis results were significant (*p* < 0.05), we conducted *post hoc* pairwise comparisons using Dunn’s test with Bonferroni adjustment to control the family-wise error rate in multiple comparisons. We used the ‘kruskal.test’ and ‘t.test’ functions from the R base package [57,58] and the ‘dunn.test’ function from the ‘dunn.test’ R package [58].

#### (iii) Elevation effect on community-level network structure

We tested whether differences in network structure between localities within each country could be explained by elevation using GLMs for each community-level network metric (*z*-score of modularity, nestedness, connectance and specialization). We used the same criteria of fitting nonlinear relationships and interactions among variables as described for the diversity metrics.

#### (iv) Effect of trait matching on species-level contribution to network structure

Finally, we evaluated whether reliance on trait matching, measured as the *z*-score of trait mismatch, influenced species-level contribution to network structure. Four generalized linear mixed-effect models (GLMMs) were performed, one for each metric as a response variable (i.e. species’ contributions to modularity, nestedness, connectance and specialization). The *z*-score of trait mismatch served as the primary predictor, along with species group (hummingbird or plant), country and elevation. Interaction terms between the fixed effects were tested to assess whether the effect of trait matching on network metrics was contingent upon country, group or elevation. If interactions were not significant, they were simplified to additive terms for model simplicity. Random intercepts were included to account for variation among species and sites. We used a *t*-distribution (Student’s *t*-distribution) error family with an identity link function, which is more robust to heavy-tailed data than the Gaussian distribution. The *t*-distribution was particularly suitable for our analysis because it handles outliers and extreme values more effectively than the classical Gaussian distribution [59]. This was particularly important since our response variables, represented as *z*-scores, were stochastic around zero and reflected stronger underlying mechanisms at the extremes, making those values critical for understanding the patterns in our data.

All models described in the statistical analyses section are reported in electronic supplementary material Annex I, table S2. All models were fitted using the ‘glmmTMB’ function in the ‘glmmTMB’ R package [60,61]. We ensured all model assumptions were satisfied by examining QQ normality plots, ordered residuals and tests for normality, overdispersion and outliers. These checks were conducted using functions available in the ‘DHARMa’ R package [61].

## 3. Results

### (a) Diversity changes across elevation

**Figure 2.**
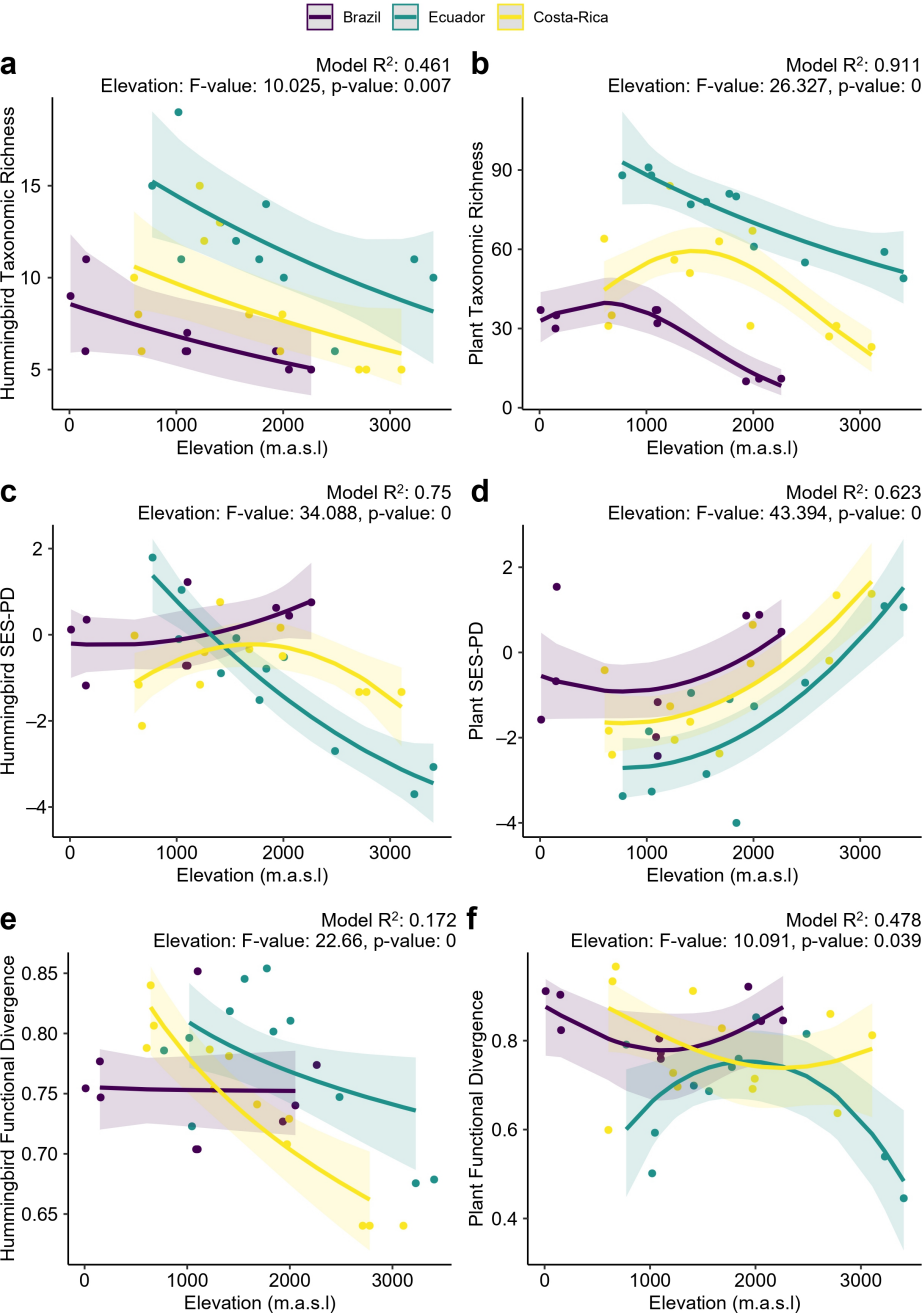
Diversity patterns across elevation gradients: taxonomic (a,b), phylogenetic (c,d) and functional (e,f). Each panel includes *R*^2^ values and ANOVA type II results for elevation (*F*-values and *p*-values), with *p*-values lower than 0.001 set to 0.

Taxonomic, phylogenetic and functional diversity varied across elevations for both hummingbirds and plants. Taxonomic diversity generally decreased with elevation for both groups, except for plant taxonomic diversity in Costa Rica, which peaked at mid-elevation (figure 2a,b). The phylogenetic diversity of hummingbirds showed country-specific trends: it decreased in Ecuador and followed a hump-shaped course in Costa Rica (figure 2c). In contrast, the phylogenetic diversity of plants tended to increase with elevation (figure 2d). Functional diversity consistently declined with elevation for hummingbirds in Ecuador and Costa Rica but not in Brazil (figure 2e). Plant functional diversity peaked at mid-elevations in Ecuador, declining at both lower and higher extremes. No clear trends were observed in the other two countries that showed weak U-shapes (figure 2f).

### (b) General patterns in community-level network structure

**Figure 3.**
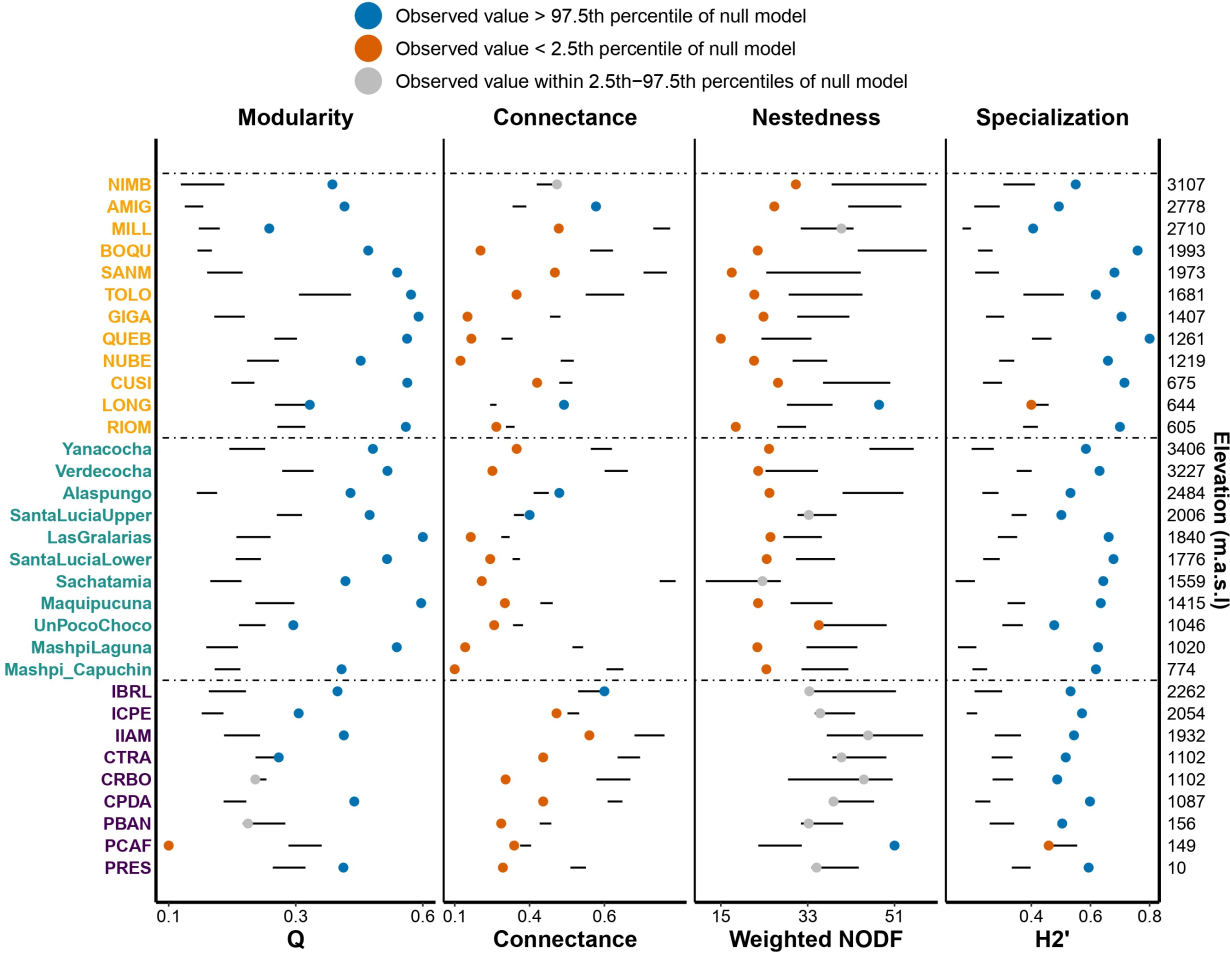
Network metrics (modularity, connectance, nestedness and community specialization) across 32 sites. Sites in Costa Rica, Ecuador and Brazil are shown in yellow, green and purple, respectively, ordered by elevation (secondary *y*-axis on the right). Each dot represents a site’s observed metric value, with horizontal black lines indicating the 95% CIs of null model expectations. Dot colours reflect the degree of deviation from null expectations, as detailed in the figure.

We found that network structure was relatively consistent across sites and countries. The *t*‐test showed that *z*-scores of network metrics significantly differed from zero, with networks being more modular (*z*-modularity = 14.891, *t* = 7.184, 95% CI = [10.663, 19.118], *p* < 0.001) and specialized (*z*-specialization = 18.747, *t* = 9.788, 95% CI = [14.841, 22.653], *p* < 0.001) and less nested (*z*-nestedness = −2.781, *t* = −3.908, 95% CI = [−4.233, −1.330], *p* < 0.001) and connected (*z*-connectance = −11.859, *t* = −4.131, 95% CI = [−17.714, −6.004], *p* < 0.001) than expected by chance. Despite these general patterns, there was still some variation among sites (figure 3).

**Figure 4.**
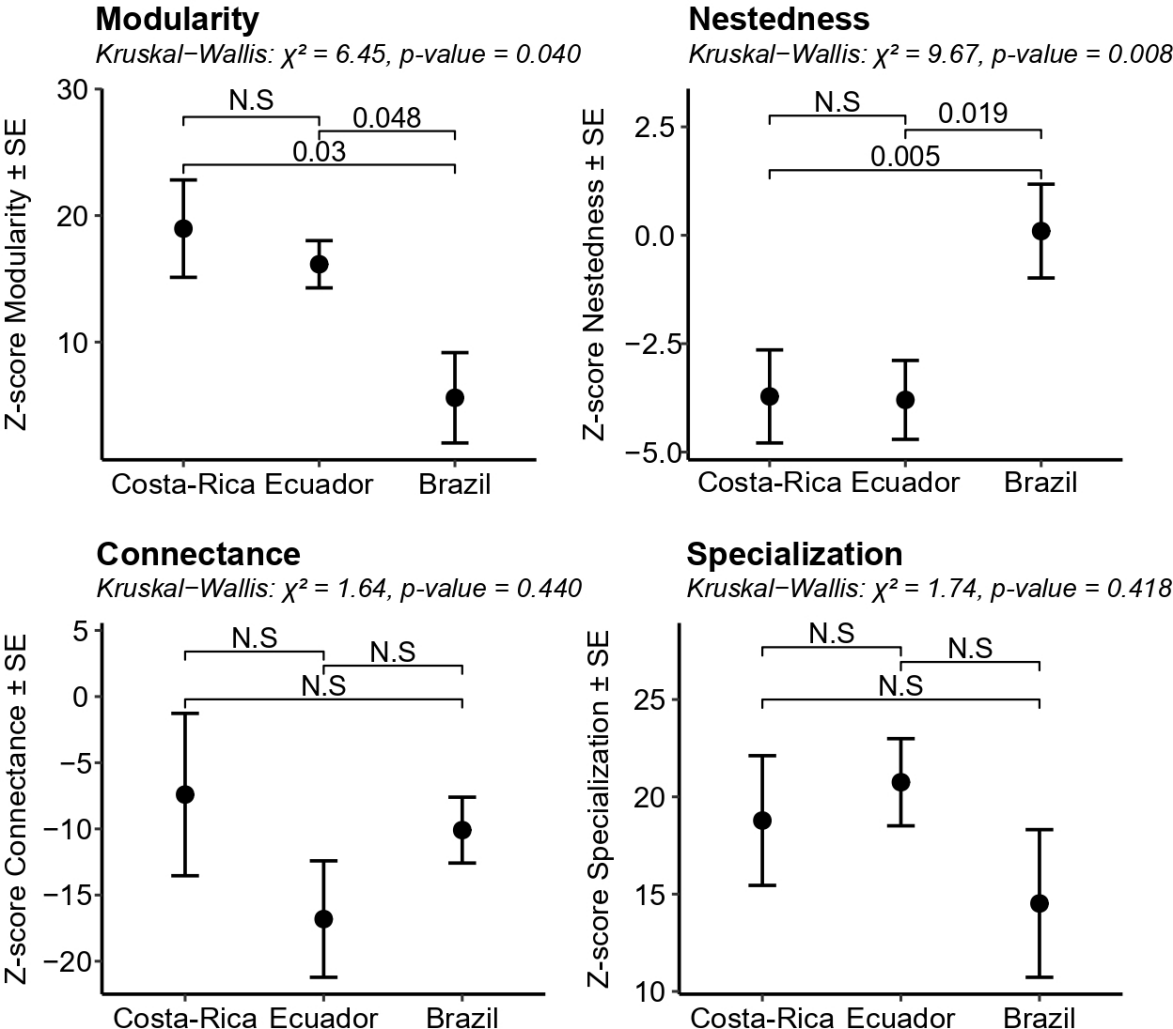
*Z*-scores of network metrics by country (mean ± s.e.). Kruskal–Wallis tests (*p* < 0.05) indicate significant differences among countries. Horizontal bars show *post hoc* pairwise comparisons (Dunn’s test), with NS indicating *p* > 0.1.

We found no differences among countries for connectance and specialization. However, modularity and nestedness differed significantly among countries (figure 4). Brazil had lower modularity than Ecuador and Costa Rica. Nestedness in Brazil fell mostly within null expectations (figure 3), indicating no deviation from random structure, whereas in Ecuador and Costa Rica, nestedness was consistently lower than expected (figures 3 and 4).

### (c) Community-level network structure variation across elevation

**Figure 5.**
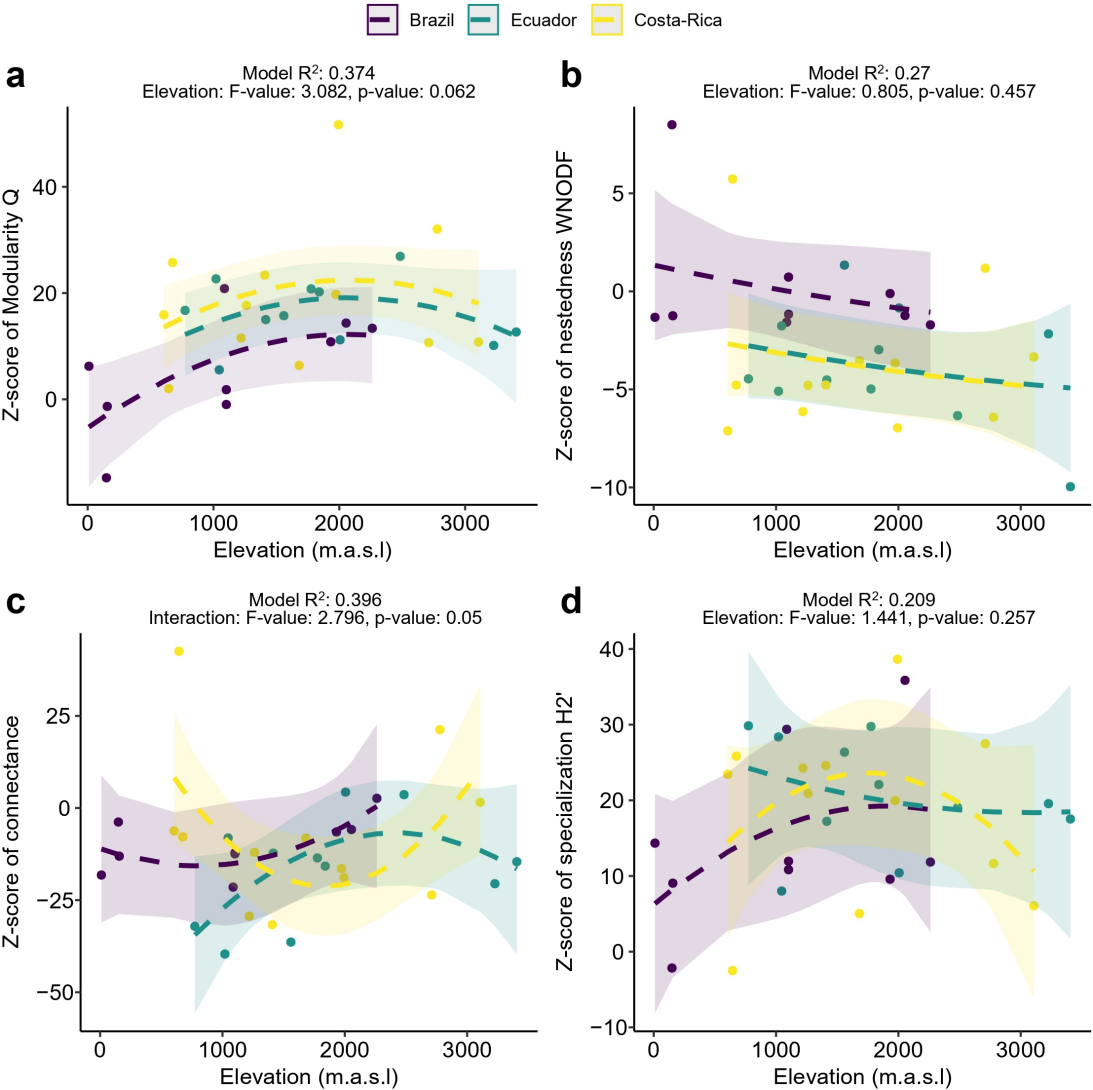
Relationships between elevation and the *z*-score of (a) modularity, (b) nestedness, (c) connectance and (d) specialization. Lines represent model predictions for each country. Each panel includes *R*² values and ANOVA Type II results for elevation (*F*-values and *p*-values). Dotted lines are used for trends with *p*-values ≥ 0.05.

Although all diversity components varied with elevation, there was no evidence of it having an influence on network structure. No significant correlations were found between elevation and nestedness or specialization (figure 5b,d), while its associations with modularity and connectance were weak and inconsistent. Modularity exhibited a marginally significant hump-shaped pattern (figure 5a). Meanwhile, connectance exhibited marginally significant trends that varied across countries (figure 5c): it followed a U-shaped curve in Costa Rica and Brazil, whereas in Ecuador, it displayed a hump-shaped pattern.

### (d) Effect of trait matching on species-level contributions to network structure

**Figure 6.**
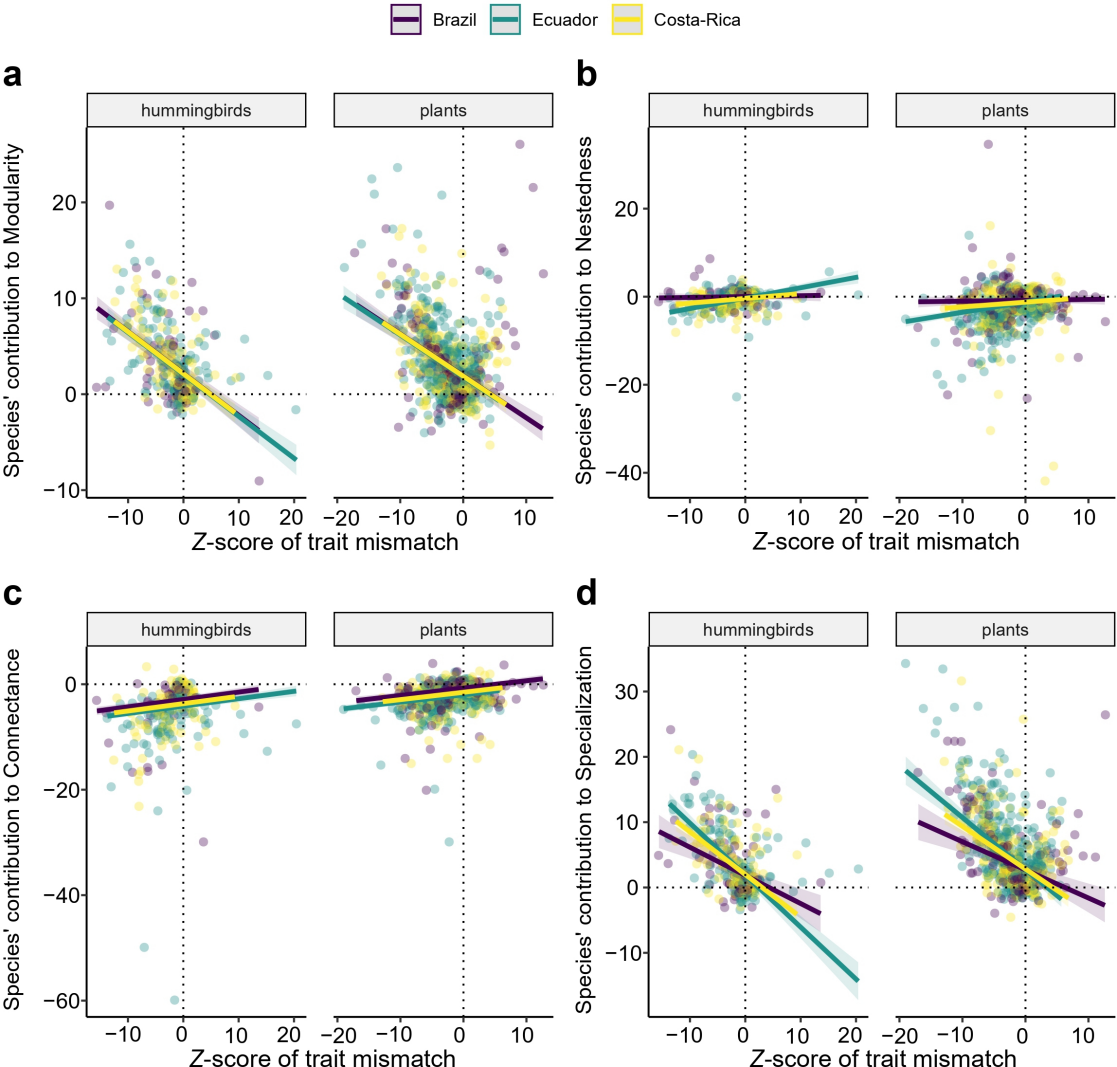
Relationship between the *z*-score of trait mismatch and species-level contributions to (a) modularity, (b) nestedness, (c) connectance and (d) specialization. A lower *z*-score of trait mismatch suggests that trait matching influences species interactions more strongly. Values around 0 indicate stochastic patterns. Positive values reflect interactions between species with significant trait mismatches.

Trait matching predicted the structure of plant–hummingbird networks. We found predominantly negative *z*-scores for trait mismatch, indicating that species tended to interact with partners that closely match their pollination traits (*t*‐test; mean = −2.286, *t* = −18.091, 95% CI [−2.534, −2.038], *p* < 0.001). The *z*-scores of trait mismatch predicted species’ contributions to network structure. Species with lower *z*-scores of trait mismatch contributed positively to modularity (*χ*² = 171.444, *p* < 0.001; figure 6a) and specialization (*χ*² = 12.037, p 0.002; figure 6d), but negatively to nestedness (*χ*² = 21.138, *p* < 0.001; figure 6b) and connectance (*χ*² = 57.769, *p* < 0.001; figure 6c). Full ANOVA results, including the effects of country, group and elevation, are available in electronic supplementary material Annex I, table S3.

## 4. Discussion

The structure of plant–hummingbird pollination networks remained relatively invariant along elevation gradients across biogeographic regions, even though taxonomic, functional and phylogenetic diversity varied considerably. Our results are consistent with studies from different taxonomic groups showing that network metrics are relatively consistent despite changes in diversity across gradients [21–23,62]. However, counter-examples are also common [7,8,18]. By attributing network structure to a specific mechanism—trait matching—that acts consistently across networks, we provide an explanation of why consistency in network structure is observed. Species whose interactions are shaped by trait matching contribute to high modularity and specialization and to low nestedness and connectance. More generally, our results suggest that consistent network structures may arise from underlying mechanisms that shape species’ interactions. Exploring these mechanisms across different diversity gradients may allow us to reconcile contrasting empirical findings.

Elevational gradients are ideal for exploring the consistency of trait-based mechanisms in determining network structure, because species, traits and evolutionary lineages involved in interactions vary across elevation. We find that taxonomic diversity generally declined with elevation for both hummingbirds and plants, although the latter often peaked at mid-elevations. These patterns are consistent with findings from many elevational gradient studies [63–65]. Phylogenetic and functional components had more complex and region-specific responses. Plant phylogenetic diversity consistently increased with elevation across countries. High elevations may favour phylogenetic diversity by incorporating lineages that migrated from colder regions and are evolutionarily distant from those that diversified *in situ* [66,67]. In contrast, hummingbird phylogenetic diversity showed country-specific trends. In Ecuador, the phylogenetic diversity of hummingbirds declined at higher altitudes, probably because only two lineages (Brilliants and Coquettes) have overcome the metabolic and aerodynamic challenges of high altitudes, leading to phylogenetic clustering [43,68]. Brazil and Costa Rica showed hump-shaped relationships between phylogenetic diversity and elevation, which may be explained by the mixing of high- and low-elevation lineages at mid-elevations. Hummingbird functional divergence decreased with elevation in Ecuador and Costa Rica and showed no discernible pattern in Brazil. In contrast, plant functional divergence showed country-specific trends, suggesting that the observed reduction in taxonomic richness affects trait distributions differently across sites.

Despite the strong influence of elevation on diversity, network metrics were similar along the elevational gradients in the three regions. Connectance and modularity showed marginally significant relationships with elevation, with connectance varying inconsistently among countries and modularity following a consistent hump-shaped pattern. Specialization and nestedness did not vary with elevation. Networks generally tended to be more modular and specialized, and less nested and connected than expected by chance. These results agree with comparisons of plant–hummingbird networks compiled from multiple sites across the Americas [69–71]. High modular and specialized networks are likely to support coevolutionary dynamics [3–5,72,73] and, in combination with low nestedness, may contribute to network stability by buffering disturbances following species loss [72,73].

We identified a key and consistent mechanism that underlies the structure of plant–hummingbird networks: trait matching. This mechanism has been documented in multiple studies [11–13,26]. We found that species with low *z*-scores of trait mismatch (i.e. species that interact with well-matched partners) were the main contributors to the high values of modularity and specialization and the low levels of nestedness and connectance observed in the networks. This pattern was reflected in the network structure: species relying on trait matching tended to form clusters with well-matched partners, leading to high modularity and specialization. This compartmentalization reduced connectance by limiting links among modules. Likewise, nestedness was low because specialists primarily interacted with other specialists within modules, rather than sharing partners with generalists. These cascading effects illustrate that network metrics are not orthogonal but capture interrelated yet complementary facets of community organization. By linking a specific mechanism to the different aspects of community organization captured by network metrics, we move beyond describing patterns to understanding the processes that shape network structure and their implications for community stability.

Our results from these three regions suggest that biogeographic contingencies influence the importance of trait-matching in structuring networks. Costa Rica and Ecuador had higher modularity and lower nestedness compared with Brazil, consistent with a stronger reliance on trait matching in these regions. The northwestern Andes and the coastal mountains of Costa Rica lie at lower latitudes, attain higher elevations and exhibit greater topographic complexity than the mountains of Brazil’s Atlantic Forest. Such conditions promote fine-scale niche partitioning, fostering greater species richness and the diversification of montane clades, such as Coquettes and Brilliants in the Andes, and Mountain Gems in Central America [74,75]. In low-latitude regions, the combination of climatic stability and steep elevational gradients facilitates short-range tracking of climatic optima, promoting species persistence and co-evolution with local floras [69,76]. Coevolution probably strengthens trait matching, explaining our observed regional differences in network structure, which agrees with evidence for stronger matching towards lower latitudes [12]. These hypotheses could be tested in species-poor and recently colonized continental regions, such as North America or dry biomes (e.g. Cerrado, Catinga), where the importance of trait matching in structuring communities may be lower. Addressing these questions could help reconcile discrepancies among empirical studies from different regions.

In conclusion, the observed diversity changes across elevation gradients and among countries do not correspond to shifts in network structure, despite some differences in the magnitude of the metrics. High modularity, specialization, low nestedness and low connectance persist across networks. This consistency appears to be driven by species-level trait matching, with species exhibiting the highest trait matching contributing the most to network structure metric patterns. Our results suggest that trait matching is a key mechanism underlying the invariant structure of plant–hummingbird networks, persisting despite profound shifts in diversity across biogeographical regions and elevational gradients. Beyond plant–hummingbird interactions, our results highlight the importance of adopting a more mechanistic approach to the study of ecological networks. Pinpointing whether trait-based mechanisms, context-dependent factors, or their combination drive interactions is key across systems and reveals the consistency of evolutionary processes in different contexts.

## Data Availability

The data and code required to replicate this research are available at [77]. Supplementary material is available online [78].

## References

[1] Bascompte J, Jordano P. 2007 Plant-animal mutualistic networks: the architecture of biodiversity. Annu. Rev. Ecol. Evol. Syst. **38**, 567–593. (10.1146/annurev.ecolsys.38.091206.095818)

[2] Bascompte J. 2010 Structure and dynamics of ecological networks. Science **329**, 765–766. (10.1126/science.1194255) 20705836

[3] Guimarães PR, Rico-Gray V, Oliveira PS, Izzo TJ, dos Reis SF, Thompson JN. 2007 Interaction intimacy affects structure and coevolutionary dynamics in mutualistic networks. Curr. Biol. **17**, 1797–1803. (10.1016/j.cub.2007.09.059) 17949981

[4] Medeiros LP, Garcia G, Thompson JN, Guimarães PR. 2018 The geographic mosaic of coevolution in mutualistic networks. Proc. Natl Acad. Sci. USA **115**, 12017–12022. (10.1073/pnas.1809088115) 30404910 PMC6255164

[5] Thompson JN. 2005 The geographic mosaic of coevolution. Chicago, IL: University of Chicago Press.

[6] Nuismer SL, Jordano P, Bascompte J. 2013 Coevolution and the architecture of mutualistic networks. Evolution **67**, 338–354. (10.1111/j.1558-5646.2012.01801.x) 23356608

[7] Dzekashu FF, Pirk CWW, Yusuf AA, Classen A, Kiatoko N, Steffan‐Dewenter I, Peters MK, Lattorff HMG. 2023 Seasonal and elevational changes of plant‐pollinator interaction networks in East African mountains. Ecol. Evol. **13**, e10060. (10.1002/ece3.10060) 37187966 PMC10175727

[8] Hoiss B, Krauss J, Steffan‐Dewenter I. 2015 Interactive effects of elevation, species richness and extreme climatic events on plant–pollinator networks. Glob. Chang. Biol. **21**, 4086–4097. (10.1111/gcb.12968) 26332102

[9] Gómez-Martínez C, González-Estévez MA, Cursach J, Lázaro A. 2022 Pollinator richness, pollination networks, and diet adjustment along local and landscape gradients of resource diversity. Ecol. Appl. **32**, e2634. (10.1002/eap.2634) 35403772 PMC9539497

[10] Bartomeus I, Gravel D, Tylianakis JM, Aizen MA, Dickie IA, Bernard‐Verdier M. 2016 A common framework for identifying linkage rules across different types of interactions. Funct. Ecol. **30**, 1894–1903. (10.1111/1365-2435.12666)

[11] Dalsgaard B *et al*. 2021 The influence of biogeographical and evolutionary histories on morphological trait‐matching and resource specialization in mutualistic hummingbird–plant networks. Funct. Ecol. **35**, 1120–1133. (10.1111/1365-2435.13784)

[12] Sonne J *et al*. 2020 Ecological mechanisms explaining interactions within plant–hummingbird networks: morphological matching increases towards lower latitudes. Proc. R. Soc. B **287**, 20192873. (10.1098/rspb.2019.2873) PMC7126064 32156208

[13] Weinstein BG, Graham CH. 2017 Persistent bill and corolla matching despite shifting temporal resources in tropical hummingbird‐plant interactions. Ecol. Lett. **20**, 326–335. (10.1111/ele.12730) 28150364

[14] Olesen JM, Jordano P. 2002 Geographic patterns in plant–pollinator mutualistic networks. Ecology **83**, 2416–2424. (10.1890/0012-9658(2002)083)

[15] Jordano P. 1987 Patterns of mutualistic interactions in pollination and seed dispersal: connectance, dependence asymmetries, and coevolution. Am. Nat. **129**, 657–677. (10.1086/284665)

[16] Schleuning M *et al*. 2012 Specialization of mutualistic interaction networks decreases toward tropical latitudes. Curr. Biol. **22**, 1925–1931. (10.1016/j.cub.2012.08.015) 22981771

[17] Chamberlain SA, Bronstein JL, Rudgers JA. 2014 How context dependent are species interactions? Ecol. Lett. **17**, 881–890. (10.1111/ele.12279) 24735225

[18] Maruyama PK *et al*. 2018 Functional diversity mediates macroecological variation in plant–hummingbird interaction networks. Glob. Ecol. Biogeogr. **27**, 1186–1199. (10.1111/geb.12776)

[19] Montoya D, Yallop ML, Memmott J. 2015 Functional group diversity increases with modularity in complex food webs. Nat. Commun. **6**, 7379. (10.1038/ncomms8379) 26059871 PMC4490355

[20] Dáttilo W, Guimarães PR, Izzo TJ. 2013 Spatial structure of ant–plant mutualistic networks. Oikos **122**, 1643–1648. (10.1111/j.1600-0706.2013.00562.x)

[21] Pérez‐Ortega S, Verdú M, Garrido‐Benavent I, Rabasa S, Green TGA, Sancho LG, de los Ríos A. 2023 Invariant properties of mycobiont‐photobiont networks in Antarctic lichens. Glob. Ecol. Biogeogr. **32**, 2033–2046. (10.1111/geb.13744)

[22] Carstensen DW, Sabatino M, Morellato LPC. 2016 Modularity, pollination systems, and interaction turnover in plant‐pollinator networks across space. Ecology **97**, 1298–1306. (10.1890/15-0830.1) 27349105

[23] Dupont YL, Padrón B, Olesen JM, Petanidou T. 2009 Spatio‐temporal variation in the structure of pollination networks. Oikos **118**, 1261–1269. (10.1111/j.1600-0706.2009.17594.x)

[24] Duchenne F *et al*. 2025 A probabilistic view of forbidden links: their prevalence and their consequences for the robustness of plant–hummingbird communities. Ecol. Lett. **28**, e70073. (10.1111/ele.70073) 39873403

[25] Bustos A, Wüest RO, Graham CH, Varassin IG. 2023 The effect of species role and trait-matching on plant fitness in a plant-hummingbird interaction network. Flora **305**, 152348. (10.1016/j.flora.2023.152348)

[26] Maglianesi MA, Blüthgen N, Böhning-Gaese K, Schleuning M. 2014 Morphological traits determine specialization and resource use in plant–hummingbird networks in the neotropics. Ecology **95**, 3325–3334. (10.1890/13-2261.1)

[27] Temeles EJ, Koulouris CR, Sander SE, Kress WJ. 2009 Effect of flower shape and size on foraging performance and trade‐offs in a tropical hummingbird. Ecology **90**, 1147–1161. (10.1890/08-0695.1) 19537537

[28] Feinsinger P. 1976 Organization of a tropical guild of nectarivorous birds. Ecol. Monogr. **46**, 257–291. (10.2307/1942255)

[29] Sánchez‐Martín R, Barreto E, Duchenne F, Varassin IG, Maglianesi M, Tinoco B, Graham CH. 2025 Functional generalism in plant–hummingbird interactions: causes and consequences from a plant perspective. Funct. Ecol. 1–12. (10.1111/1365-2435.70167)

[30] Guevara EA, Bello C, Poveda C, McFadden IR, Schleuning M, Pellissier L, Graham CH. 2023 Hummingbird community structure and nectar resources modulate the response of interspecific competition to forest conversion. Oecologia **201**, 761–770. (10.1007/s00442-023-05330-z) 36754882 PMC10038955

[31] Leimberger KG, Dalsgaard B, Tobias JA, Wolf C, Betts MG. 2022 The evolution, ecology, and conservation of hummingbirds and their interactions with flowering plants. Biol. Rev. **97**, 923–959. (10.1111/brv.12828) 35029017

[32] Martín González AM *et al*. 2015 The macroecology of phylogenetically structured hummingbird–plant networks. Glob. Ecol. Biogeogr. **24**, 1212–1224. (10.1111/geb.12355)

[33] Verdú M *et al*. 2023 RecruitNet: a global database of plant recruitment networks. Ecology **104**, e3923. (10.1002/ecy.3923) 36428233 PMC10078134

[34] Botella C *et al*. 2024 Land‐use intensity influences European tetrapod food webs. Glob. Chang. Biol. **30**, e17167. (10.1111/gcb.17167) 38348640

[35] Brimacombe C, Bodner K, Fortin MJ. 2024 Applying a method before its proof of concept: a cautionary tale using inferred food webs. Glob. Chang. Biol. **30**, e17360. (10.1111/gcb.17360) 38822572

[36] Santander T *et al*. 2025 Plant–hummingbird interactions, floral abundance and floral traits for three altitudinal gradients in the Americas (Under review).

[37] Maruyama PK, Vizentin‐Bugoni J, Oliveira GM, Oliveira PE, Dalsgaard B. 2014 Morphological and spatio‐temporal mismatches shape a neotropical savanna plant‐hummingbird network. Biotropica **46**, 740–747. (10.1111/btp.12170)

[38] Faegri K, van der Pijl L. 1979 Principles of pollination ecology. Oxford, UK; New York, NY: Pergamon Press. (10.1016/B978-0-08-023160-0.50020-7)

[39] Weinstein BG. 2018 Scene‐specific convolutional neural networks for video‐based biodiversity detection. Methods Ecol. Evol. **9**, 1435–1441. (10.1111/2041-210x.13011)

[40] Schneider CA, Rasband WS, Eliceiri KW. 2012 NIH Image to ImageJ: 25 years of image analysis. Nat. Methods **9**, 671–675. (10.1038/nmeth.2089) 22930834 PMC5554542

[41] Skandalis DA *et al*. 2017 The biomechanical origin of extreme wing allometry in hummingbirds. Nat. Commun. **8**, 1047. (10.1038/s41467-017-01223-x) 29051535 PMC5715027

[42] Dakin R, Segre PS, Straw AD, Altshuler DL. 2018 Morphology, muscle capacity, skill, and maneuvering ability in hummingbirds. Science **359**, 653–657. (10.1126/science.aao7104) 29439237

[43] Graham CH, Parra JL, Tinoco BA, Stiles FG, McGuire JA. 2012 Untangling the influence of ecological and evolutionary factors on trait variation across hummingbird assemblages. Ecology **93**, S99–S111. (10.1890/11-0493.1)

[44] Tobias JA *et al*. 2022 AVONET: morphological, ecological and geographical data for all birds. Ecol. Lett. **25**, 581–597. (10.1111/ele.13898) 35199922

[45] Zanata TB *et al*. 2017 Global patterns of interaction specialization in bird–flower networks. J. Biogeogr. **44**, 1891–1910. (10.1111/jbi.13045)

[46] Kembel SW, Cowan PD, Helmus MR, Cornwell WK, Morlon H, Ackerly DD, Blomberg SP, Webb CO. 2010 Picante: R tools for integrating phylogenies and ecology. Bioinformatics **26**, 1463–1464. (10.1093/bioinformatics/btq166) 20395285

[47] Laliberté E, Legendre P. 2010 A distance‐based framework for measuring functional diversity from multiple traits. Ecology **91**, 299–305. (10.1890/08-2244.1) 20380219

[48] Villéger S, Mason NWH, Mouillot D. 2008 New multidimensional functional diversity indices for a multifaceted framework in functional ecology. Ecology **89**, 2290–2301. (10.1890/07-1206.1) 18724739

[49] Beckett SJ. 2016 Improved community detection in weighted bipartite networks. R. Soc. Open Sci. **3**, 140536. (10.1098/rsos.140536) 26909160 PMC4736915

[50] Dunne JA, Williams RJ, Martinez ND. 2002 Food-web structure and network theory: the role of connectance and size. Proc. Natl Acad. Sci. USA **99**, 12917–12922. (10.1073/pnas.192407699) 12235364 PMC130560

[51] Blüthgen N, Menzel F, Blüthgen N. 2006 Measuring specialization in species interaction networks. BMC Ecol. **6**, 9. (10.1186/1472-6785-6-9) 16907983 PMC1570337

[52] Almeida-Neto M, Ulrich W. 2011 A straightforward computational approach for measuring nestedness using quantitative matrices. Environ. Model. Softw. **26**, 173–178. (10.1016/j.envsoft.2010.08.003)

[53] Dormann CF, Frund J, Bluthgen N, Gruber B. 2009 Indices, graphs and null models: analyzing bipartite ecological networks. Open Ecol. J. **2**, 7–24. (10.2174/1874213000902010007)

[54] Bascompte J, Jordano P, Olesen JM. 2006 Asymmetric coevolutionary networks facilitate biodiversity maintenance. Science **312**, 431–433. (10.1126/science.1123412) 16627742

[55] Coux C, Rader R, Bartomeus I, Tylianakis JM. 2016 Linking species functional roles to their network roles. Ecol. Lett. **19**, 762–770. (10.1111/ele.12612) 27169359

[56] Saavedra S, Stouffer DB, Uzzi B, Bascompte J. 2011 Strong contributors to network persistence are the most vulnerable to extinction. Nature **478**, 233–235. (10.1038/nature10433) 21918515

[57] R Core Team. 2024 R: A language and environment for statistical computing. Vienna, Austria: R Foundation for Statistical Computing.

[58] Dinno A. 2024 dunn.test: Dunn’s test of multiple comparisons using rank sums. See https://cran-e.com/package/dunn.test.

[59] Lange KL, Little RJA, Taylor JMG. 1989 Robust statistical modeling using the t distribution. J. Am. Stat. Assoc. **84**, 881–896. (10.1080/01621459.1989.10478852)

[60] Brooks M, Kristensen K, van Benthem K, Magnusson A, Berg C, Nielsen A, Skaug H, Mächler M, Bolker B. 2017 glmmTMB balances speed and flexibility among packages for zero-inflated generalized linear mixed modeling. R J. **9**, 378–400. (10.32614/RJ-2017-066)

[61] Hartig F. 2022 Package ‘DHARMa’ title residual diagnostics for hierarchical (multi-level / mixed) regression models. Cran. See https://cran.r-project.org/web/packages/DHARMa/vignettes/DHARMa.html.

[62] Jordano P, Bascompte J, Olesen JM. 2003 Invariant properties in coevolutionary networks of plant–animal interactions. Ecol. Lett. **6**, 69–81. (10.1046/j.1461-0248.2003.00403.x)

[63] Rahbek C. 1995 The elevational gradient of species richness: a uniform pattern? Ecography **18**, 200–205. (10.1111/j.1600-0587.1995.tb00341.x)

[64] Sandoya V, Pauchard A, Cavieres LA. 2017 Natives and non‐natives plants show different responses to elevation and disturbance on the tropical high Andes of Ecuador. Ecol. Evol. **7**, 7909–7919. (10.1002/ece3.3270) 29043044 PMC5632626

[65] Galván-Cisneros CM, Villa PM, Coelho AJP, Campos PV, Meira-Neto JAA. 2023 Altitude as environmental filtering influencing phylogenetic diversity and species richness of plants in tropical mountains. J. Mt. Sci. **20**, 285–298. (10.1007/s11629-022-7687-9)

[66] van der Hammen T. 1974 The pleistocene changes of vegetation and climate in tropical South America. J. Biogeogr **1**, 3–26. (10.2307/3038066)

[67] González-Caro S, Umaña MN, Álvarez E, Stevenson PR, Swenson NG. 2014 Phylogenetic alpha and beta diversity in tropical tree assemblages along regional-scale environmental gradients in northwest South America. J. Plant Ecol. **7**, 145–153. (10.1093/jpe/rtt076)

[68] Graham CH, Parra JL, Rahbek C, McGuire JA. 2009 Phylogenetic structure in tropical hummingbird communities. Proc. Natl Acad. Sci. USA **106**, 19673–19678. (10.1073/pnas.0901649106) 19805042 PMC2780942

[69] Dalsgaard B *et al*. 2011 Specialization in plant-hummingbird networks is associated with species richness, contemporary precipitation and quaternary climate-change velocity. PLoS One **6**, e25891. (10.1371/journal.pone.0025891) 21998716 PMC3187835

[70] Vizentin-Bugoni J, Maruyama PK, Sazima M. 2014 Processes entangling interactions in communities: forbidden links are more important than abundance in a hummingbird–plant network. Proc. R. Soc. B **281**, 20132397. (10.1098/rspb.2013.2397) PMC4027382 24552835

[71] Vizentin‐Bugoni J, Maruyama PK, Debastiani VJ, Duarte L da S, Dalsgaard B, Sazima M. 2016 Influences of sampling effort on detected patterns and structuring processes of a neotropical plant–hummingbird network. J. Anim. Ecol. **85**, 262–272. (10.1111/1365-2656.12459) 26476103

[72] Krause AE, Frank KA, Mason DM, Ulanowicz RE, Taylor WW. 2003 Compartments revealed in food-web structure. Nature **426**, 282–285. (10.1038/nature02115) 14628050

[73] Yan C. 2022 Nestedness interacts with subnetwork structures and interconnection patterns to affect community dynamics in ecological multilayer networks. J. Anim. Ecol. **91**, 738–751. (10.1111/1365-2656.13665) 35061910

[74] McGuire JA, Witt CC, Remsen JV, Corl A, Rabosky DL, Altshuler DL, Dudley R. 2014 Molecular phylogenetics and the diversification of hummingbirds. Curr. Biol. **24**, 910–916. (10.1016/j.cub.2014.03.016) 24704078

[75] Bleiweiss R. 1998 Origin of hummingbird faunas. Biol. J. Linn. Soc. **65**, 77–97. (10.1111/j.1095-8312.1998.tb00352.x)

[76] Sandel B, Arge L, Dalsgaard B, Davies RG, Gaston KJ, Sutherland WJ, Svenning JC. 2011 The influence of late quaternary climate-change velocity on species endemism. Science **334**, 660–664. (10.1126/science.1210173) 21979937

[77] Sánchez-Martín R, Graham C. 2025 Data and R code for: Mechanisms influencing network topology in plant–hummingbird pollination networks. Zenodo. (10.5281/zenodo.15180374)41290172

[78] Sánchez-Martín R, Barreto E, Maxwell MF, Duchenne F, Beck H, Bobato R *et al*. 2025 Supplementary material from: Mechanisms influencing network topology in plant-hummingbird pollination networks. Figshare. (10.6084/m9.figshare.c.8124505)41290172

